# Syphilis

**Published:** 1887-12

**Authors:** 


*Syphilis.* By JONATHAN HUTCHINSON, F.R.S. Pp. 532. London: CASSELL & CO. 1887.

A book by Jonathan Hutchinson on Syphilis must be welcome to the whole profession. Whether the welcome would not have been more hearty had the book been framed on broader lines than those laid out for Cassell's *Clinical Manuals*, we can scarcely venture to say. In any case there can be no doubt as to its hearty reception by all students of syphilis.

The first thing that strikes one is the peculiar arrangement of the book. The author says in the Preface, "I have aimed less at systematic completeness than at clinical exposition;" and herein he doubtless follows the natural and well-known bent of his own mind. But when we note that the systematic description occupies only 92 pages, and commentaries or illustrative cases fill the rest of the book, we can scarcely help doubting whether the balance of material is evenly divided. It seems to us that systematic description is too short, and clinical illustrations too long. The main purpose of a systematic medical work is to generalise from cases—to eliminate the unessential, and isolate and dwell upon the essential, features of a disease. Unessential, rare, and curious features are liable to be raised to undue importance at the expense of the essential and common features, if individual cases are too much dwelt upon. We all have our cases; he who has the most philosophic mind generalises from them. One case does not prove a hypothesis, though it may illustrate a principle. When a general principle has been established, a case quoted may give concreteness and vitality to the narrative; it is treading on uncertain ground to answer an important query by the record of a single case.

It is often a fault in small works written by great authorities that they take too much for granted in the reader's knowledge. The learned author dwells unduly on those difficult and abstruse questions which exercise his own mind, and skims over or ignores the elemental facts. There is just a little of this in the work. For instance, the first sentence in the first chapter reads: "The first step, in order that we should understand syphilis, is to recognise that it is by no means necessarily a venereal disease." In the same paragraph we are told that, "in practice we meet with *numerous* cases in which the contagion takes place upon parts distant from the genitals; on the finger, as *so often* seen in the case of medical men and midwives; on the lips, . . . . ; in the sores produced by vaccination." In the whole chapter there is not a single definite statement that syphilis is a venereal disease. And surely it is the exception to meet with primary sores away from the genitals, and sores from vaccination are certainly not common. This example will show the peculiarities of the book which we venture to criticise.

As a whole the work is one of extreme value, embodying in small space the teachings and experience of a man who has earned a just title to be considered a master on his subject.

